# Enhancing mental health pre-service training with the WHO mhGAP Intervention Guide: experiences learned and the way forward

**DOI:** 10.1186/s13034-020-00354-2

**Published:** 2021-01-11

**Authors:** Silje Akselberg Iversen, Brian Ogallo, Myron Belfer, Daniel Fung, Christina W. Hoven, Kenneth Carswell, Norbert Skokauskas

**Affiliations:** 1 Center for Child and Youth Mental Health and Child Protection, IPH, Norwegian University of Science and Technology, Trondheim, Norway; 2 Department of Mental Health and Substance Abuse, World Health Organization, Geneva, Switzerland; 3 Department of Psychiatry, Harvard Medical School, Boston, MA USA; 4 Institute of Mental Health, Singapore, Singapore; 5 The International Association for Child and Adolescent Psychiatry and Allied Profession, Geneva, Switzerland; 6 New York State Psychiatric Institute, Columbia University Medical Center, New York, NY USA; 7 The World Psychiatric Association, Child and Adolescent Psychiatry Section, Geneva, Switzerland

**Keywords:** World Health Organization, mhGAP (Mental Health Gap Action Programme), mhGAP-IG (Mental Health Gap Action Programme Intervention Guide), Child and adolescent mental health, Child and adolescent mental health services

## Abstract

There is currently a high global demand for mental health professionals, including child and adolescent mental health professionals. In 2020, the World Health Organization (WHO) published “Enhancing mental health pre-service training with the mhGAP-Intervention Guide: experiences and lessons learned” to address the proposition of implementing Mental Health Gap Action Programme Intervention Guide (mhGAP-IG) materials and principles as a component of pre-service training. By integrating the mhGAP-IG within pre-service training, future healthcare providers will acquire theoretical knowledge and early exposure to practical knowledge and will be better prepared for their future work.

Examples demonstrate that mhGAP-IG pre-service training can be successfully implemented in diverse settings and in various pre-service training programs. It can be used in small group learning activities and short courses, taught through lectures, used as a clinical tool to teach students (i.e. medical, nursing students) and medical doctors in training. We can enhance pre-service training with the mhGAP-IG and contribute to a learning environment, which nurtures knowledge and skills required to help people with mental health needs.

Globally, one-quarter of disability-adjusted life years (DALYs) are due to mental, neurological, and substance use (MNS) disorders [1]. There is currently a high global demand for mental healthcare professionals, including child and adolescent mental health professionals [2, 3]. Adjustments to academic programs need to be made to address such demands and to increase the number of mental health care providers [4].

The World Health Organization (WHO) strives to combat both communicable (i.e., COVID-19, and other diseases that are transmitted from person to person) and non-communicable diseases (i.e., diabetes, heart diseases), including mental health disorders by coordinating and supporting international health initiatives for the United Nations system [5]. One example of such an initiative is integrating mental health in general health care through the Mental Health Gap Action Programme (mhGAP) [6]. The mhGAP was launched in 2008 with the objectives and commitments of stakeholders (i.e., health services, civil society associations, governmental bodies etc.) to increase financial and human resources for the care of MNS disorders and to achieve higher coverage in low- and middle-income countries [6]. Furthermore, in 2010 the Mental Health Gap Action Programme Intervention Guide (mhGAP-IG) was developed and released to provide evidence-based guidance for the assessment and integrated management of common MNS disorders in non-specialized health settings [7].

The current version of the mhGAP-IG, released in 2016, includes information on essential care, clinical practice, and a master chart of the most common presentations of priority mental health conditions including those for child and adolescent disorders [7]. The mhGAP-IG intends to contribute towards achieving the specific goals of the WHO’s Comprehensive Mental Health Action Plan 2013–2020, including universal health coverage of MNS disorders by providing community-based, comprehensive, integrated, and responsive mental health care services [7].

Meanwhile, a systematic review published in 2018 on the implementation of the mhGAP-IG highlighted the fact that even though 100 countries have been using it, implementation was mainly in in-service training [8]. With the significant costs and resources required for in-service training, there is a need to utilize the mhGAP-IG within pre-service training [9]. Pre-service training refers to the teaching programs (e.g., at medical, public health and nursing schools) provided for future health workers before entering service roles. By integrating the mhGAP-IG within pre-service training, future healthcare providers will acquire theoretical knowledge and early exposure to practical knowledge and will be better prepared for their work. The mhGAP-IG provides an easy-to-follow approach to integrating teaching materials and can be used to train nursing, medical, social work, and psychology students before they enter their respective service roles [7].

In 2020, the WHO published “Enhancing mental health pre-service training with the mhGAP Intervention Guide: experiences and lessons learned” to address the proposition of implementing mhGAP-IG materials and principles as a component of pre-service training [9]. Prior to the development of this document, several technical consultations took place. Two meetings were held during large international congresses and one during mhGAP Forum 2018 at the WHO Headquarters. Professionals who were both responsible for and involved in pre-service mental health training discussed mhGAP-IG implementation efforts and explored future opportunities to use mhGAP in pre-service training. A common conclusion was that in-service mhGAP training can be one pillar in strengthening the health care workforce but if it is not accompanied by other measures, such as pre-service training, then the desired objective of bridging the gap will not be achieved. Subsequently, later in the same year, the first mhGAP-IG pre-service Training of Trainers and Supervisors course in child and adolescent mental health was held in Ukraine for key stakeholders and educational leaders [9].

“Enhancing mental health pre-service training with the mhGAP Intervention Guide: experiences and lessons learned” outlines principles and approaches for integrating the mhGAP-IG in a way that will fit each academic institution’s unique teaching process used by the individual staff members, administrators, and students [9]. It includes step-by-step advice to facilitate a teaching institution to take ownership of the process, enhance the curriculum with mhGAP-IG and contribute to each country’s specific mental health strategy [9]. To implement the mhGAP-IG into pre-service training, there are seven phases to consider. In the first phase, it is essential to consult with relevant stakeholders to create awareness and inform clinical educators about the principles of mhGAP-IG. For the second phase, educational leaders need to be trained with the mhGAP-IG, who will, in turn, be able to train teaching faculty staff. Moving on, in phase three, a team should be assembled to make a work plan on how to enhance the curriculum to implement the mhGAP-IG. In phase four, the current curriculum should be reviewed and enhanced accordingly, before moving on to phase five, where the mhGAP-IG should be adapted to fit the teaching institution’s circumstances. The next step is to deliver a pilot curriculum before moving on to the last step of evaluating and revising the pilot curriculum, and then implementing the revised curriculum [9].

As of today, there are already multiple examples of successful implementation of mhGAP-IG into pre-service training [9–11]. For example, Kyiv Medical University (KMU) was the first university in Ukraine to introduce the mhGAP-IG as a component of their official curriculum. The revised psychiatry curricula at KMU aims to strengthen the evidence-based teaching practices, to emphasize community-orientated mental health care, and to use interactive teaching methods, such as small group learning activities and clinical tools during placements. There is a hope that these changes at KMU will attract more students to psychiatry, and ultimately will contribute towards the reduction of the mental health treatment gap in Ukraine [10, 11].

Some countries utilized the mhGAP-IG to create completely new programmes instead of integrating it into old curricula, such as the University of Ibadan in Nigeria that established a Master of Science Programme in Child and Adolescent Mental Health, an 18-month long programme. Similar to the KMU, the University of Ibadan in Nigeria also utilized the mhGAP-IG to teach medical students about child and adolescent mental health [11].

mhGAP-IG has also been used to train junior medical doctors. For example, Universidad Nacional Autonoma de Mexico developed a 20-h course using the mhGAP-IG by revising their curriculum and adding all 10 modules of the mhGAP-IG [11]. Additionally, they initiated a mhGAP-IG pre-service training program for third-year medical students [9, 11]. mhGAP-IG has also been successfully introduced in pre-service training of nurses and paramedics. For example, the Phebe Paramedical Training Program and School of Nursing, the first and largest paramedical and nursing school in Liberia, introduced the mhGAP-IG to train nurses and paramedics. The identified benefits of mhGAP-IG pre-service training were ease of use, comprehensibility, and enhanced skills and knowledge [11].

The examples above demonstrate that mhGAP-IG pre-service training can be successfully implemented in diverse settings and various pre-service training programs. mhGAP-IG can be used in small group learning activities and short courses taught through lectures, used as a clinical tool to teach students and doctors in training [11].

“Enhancing mental health pre-service training with the mhGAP Intervention Guide: experiences and lessons learned” outlines principles and approaches for integrating the mhGAP-IG into pre-service training. There are several advantages to providing mhGAP in pre-service training, such as the benefit for people with mental health conditions by introducing future providers, early in their careers, to the practical knowledge and clinical tools needed to assess and manage such conditions. Another advantage is that with its integration into teaching curricula, there is potential to be more cost-effective as it reduces the need for further in-service training and additional resources. Last but not least, it provides a common understanding among different types of health workers and a broad range of skills to prepare for their future profession. This can strengthen health systems in the long term.

“Enhancing mental health pre-service training with the mhGAP Intervention Guide: experiences and lessons learned” may be especially helpful for stakeholders and others who are thinking about revising or introducing a new mental health curriculum. We can enhance pre-service training with the mhGAP-IG and contribute to a learning environment that nurtures knowledge and skills required to help people with mental health needs.

## Data Availability

Not applicable.

## Abbreviations

DALYS: Disability-adjusted life years
KMU: Kyiv Medical University
mhGAP-IG: mhGAP-Intervention Guide
mhGAP: Mental Health Gap Action Programme
MNS: Mental, neurological and substance use disorders
WHO: World Health Organization
